# Empagliflozin Attenuates Hyperuricemia by Upregulation of ABCG2 via AMPK/AKT/CREB Signaling Pathway in Type 2 Diabetic Mice

**DOI:** 10.7150/ijbs.33007

**Published:** 2020-01-01

**Authors:** Yun-hong Lu, Yun-peng Chang, Ting Li, Fei Han, Chun-jun Li, Xiao-yu Li, Mei Xue, Ying Cheng, Zi-yu Meng, Zhe Han, Bei Sun, Li-ming Chen

**Affiliations:** 1 NHC Key Laboratory of Hormones and Development (Tianjin Medical University), Tianjin Key Laboratory of Metabolic Diseases, Tianjin Medical University Chu Hsien-I Memorial Hospital & Tianjin Institute of Endocrinology, Tianjin 300134, China; 2 Tianjin Medical University General Hospital Airport Hospital, Tianjin 300308; 3 Department of Endocrinology and Metabolism, the Third Central Hospital of Tianjin, 83 Jintang Road, Hedong District, Tianjin 300170, China.

**Keywords:** hyperuricemia, SGLT2, AMPK, empagliflozin, ABCG2, CREB

## Abstract

Hyperuricemia (HUA) is a metabolic disease characterized by elevated serum uric acid (SUA). Empagliflozin, a kind of sodium-glucose cotransporter 2 inhibitors, has recently emerged as a new antidiabetic agent by facilitating glucose excretion in urine. Moreover, there was evidence of SUA reduction following treatment with empagliflozin in addition to glycaemic control, while the molecular mechanisms remain unknown. To investigate the potential mechanisms, the model of type 2 diabetes (T2DM) with HUA was established by combination of peritoneal injection of potassium oxonate and intragastric administration of hypoxanthine in KK-Ay mice. A series of method such as RT-PCR, western blot, immunochemistry, immunofluorescence were conducted to explore the mechanism. Our results showed that empagliflozin significantly ameliorated the levels of SUA and blood glucose in T2DM mice with HUA. Furthermore, in both kidney and ileum, empagliflozin obviously promoted protein expression of uric acid (UA) transporter ABCG2, p-AMPK, p-AKT and p-CREB. The same trend was observed in human tubular epithelial (HK-2) cells. Additionally, through application of an AMPK inhibitor (Compound C), it was further confirmed empagliflozin exerted its anti-hyperuricemic effects in an AMPK dependent manner. Meanwhile, with the help of ChIP assay and luciferase reporter gene assay, we found that CREB further activated ABCG2 via binding to the promoter of ABCG2 to induce transcription. Taken together, our study demonstrated that empagliflozin treatment played an essential role in attenuating HUA by upregulation of ABCG2 via AMPK/AKT/CREB signaling pathway.

## Introduction

Hyperuricemia (HUA) is associated with a variety of diseases such as gout and diabetes 1. There is high prevalence of HUA among patients with type 2 diabetes (T2DM). Various studies have indicated that incidence of HUA in patients with T2DM was respectively 24.1% 2 and 33.8% 3. Moreover, HUA has proven to be an independent risk factor for kidney disease progression 4, 5, and significantly correlated with the severity of retinopathy in patients with T2DM 6. Accumulating data has indicated that HUA was closely associated with risks of increased prevalence of cardiovascular disease 2, 4, carotid intima-media thickness 7, hypertension 8 and metabolic syndrome 8 in individuals with T2DM. Thus developing a new type of agent to improve hyperglycemia and HUA seems increasingly urgent.

Uric acid (UA) is a product generated from metabolism of purines by xanthine oxidase (XO). In brief, HUA occurs when the balance between production and complicated course of UA handing in renal tubules and intestines is disrupted 9. UA transporters are involved in complex processes. Urate transporter 1 (URAT1, SLC22A12) and glucose transporter 9 (GLUT9, SLC2A9) are responsible for renal reabsorption, and ATP-binding cassette subfamily G member 2 (ABCG2), organic anion transporter 1 (OAT1, SLC22A6), organic anion transporter 3 (OAT3, SLC22A8) facilitates renal secretion 9. In addition, GLUT9 and ABCG2 has been also found to be expressed in intestines, which take part in the homeostasis of UA levels as well 10.

The sodium-glucose co-transporter 2 (SGLT2) is primarily located in renal proximal tubules, and responsible for renal glucose reabsorption to maintain blood glucose homeostasis 11. SGLT2 inhibitors, which exert the effect of blocking SGLT2 to promote urinary glucose excretion, have been developed as a newly approved oral hypoglycemic drug 11. Empagliflozin, a potent and selective SGLT2 inhibitor 12, has been reported to reduce cardiovascular mortality 13, reduce blood pressure 14, and slower progression of kidney disease 15 in addition to glycemic control 16, 17. Notably, it has been reported that empagliflozin helped to reduce serum uric acid (SUA) 18, 19.

To date, the underlying mechanisms that empagliflozin reduces SUA remains largely unexplored. This study is designed to assess the role of empagliflozin on SUA in KK-Ay mice with HUA, and the underlying mechanism is involved with a focus on SGLT2 inhibitor on renal and intestinal UA transporters.

## Results

### Empagliflozin decreased serum uric acid and increased urinary uric acid excretion

**Figure 1:**
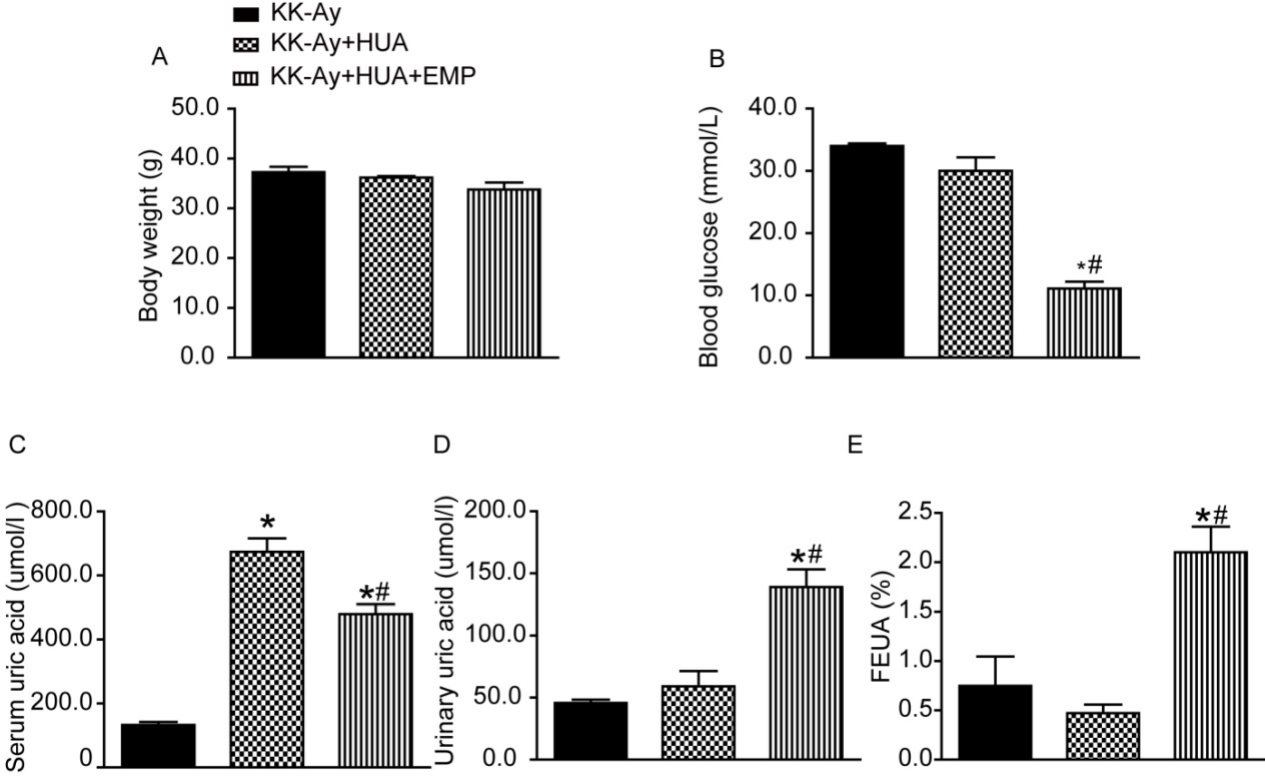
** Effects of empagliflozin on biochemical parameter alterations in KK-Ay mice with hyperuricemia**. (A-E) Body weight, blood glucose, serum uric acid, urinary uric acid and FEUA in the KK-Ay group, KK-Ay+HUA group and KK-Ay +HUA+EMP group after 8 week-treatment with empagliflozin. There was no significant difference in body weight among the three groups, while empagliflozin treatment notably lowered blood glucose and serum uric acid levels and increased the urinary uric acid excretion and FEUA. One-way ANOVA followed by Tukey's test was applied to compare the differences between any two of three groups. The data are presented as the mean ± SEM (n = 5 for each group). *P < 0.05 vs. KK-Ay group; ^#^P < 0.05 vs. KK-Ay+HUA group. KK-Ay, non-treated KK-Ay mice as control; KK-Ay+HUA, non-treated KK-Ay mice with hyperuricemia induced by combination of peritoneal injection of potassium oxonate at dose of 250mg/kg and intragastric administration of hypoxanthine at dose of 300mg/kg; KK-Ay+HUA+EMP, empagliflozin-treated KK-Ay mice with hyperuricemia induced by combination of peritoneal injection of potassium oxonate at dose of 250mg/kg and intragastric administration of hypoxanthine at dose of 300mg/kg. FEUA, fractional excretion of uric acid.

The body weight of mice from KK-Ay group, KK-Ay+HUA group, KK-Ay+HUA+EMP group was significantly increased compared with the body weight of C57BL/6J mice (Supplemental Figure 1A), while there was no significant difference in body weight among the KK-Ay group, KK-Ay+HUA group, KK-Ay+HUA+EMP group (Fig. 1A). As expected, the blood glucose in the KK-Ay group, KK-Ay+HUA group, KK-Ay+HUA+EMP group were significantly higher than that in the C57BL/6J mice (Supplemental Figure 1B), and there were no significant difference in blood glucose levels between KK-Ay group and KK-Ay+HUA group (Fig. 1B, Supplemental Figure 1B), but empagliflozin treatment notably lowered blood glucose levels (Fig. 1B, Supplemental Figure 1B). Next, we observed no notable difference in SUA between C57BL/6J group and KK-Ay group (Supplemental Figure 1C), however, the SUA of mice in KK-Ay+HUA group were dramatically increased (Fig. 1C, Supplemental Figure 1C), while empagliflozin obviously reduced the SUA (Fig. 1C, Supplemental Figure 1C). It was also demonstrated that the urinary UA levels and FEUA were significantly higher for KK-Ay+HUA+EMP group than KK-Ay group and KK-Ay+HUA group (Fig. 1D-E, Supplemental Figure 1D-E). Moreover, we found that empagliflozin increased the urine volume and urinary glucose excretion (Supplemental Figure 1F-G).

### Empagliflozin ameliorated histopathologic changes of kidney and ileum

**Figure 2:**
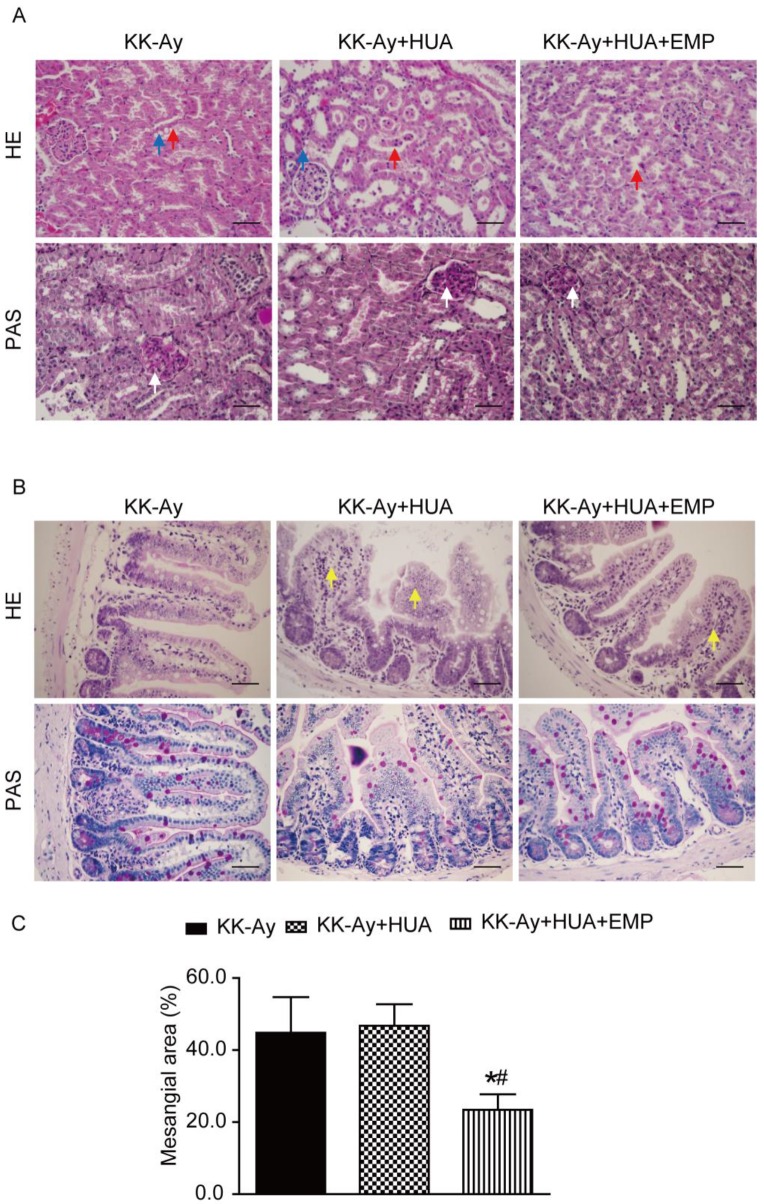
** Effects of empagliflozin on histopathologic changes of kidney and ileum from KK-Ay mice with hyperuricemia.** (A) Photomicrographs of kidney (original magnification × 400, bars = 50μm). KK-Ay+HUA group displayed tubular dilatation, hydropic degeneration and deposited mesangial matrix compared with mice in the KK-Ay group, however, those pathological features were significantly attenuated in the KK-Ay+HUA+EMP group. Red arrows indicate the tubular dilatation, blue arrows indicate the hydropic degeneration, and white arrows indicate deposited mesangial matrix. (B) Photomicrographs of ileum (original magnification × 400, bars = 50μm). Empagliflozin remarkably improved the histology of ileum in the KK-Ay +HUA+EMP group. Yellow arrows indicate abnormality of ileum. (C) Analysis of mesangial area expansion. One-way ANOVA followed by Tukey's test was applied to compare the differences between any two of three groups. The data are presented as the mean ± SEM (n = 5 for each group). ^*^P < 0.05 vs. KK-Ay group; ^#^P < 0.05 vs. KK-Ay+HUA group. KK-Ay, non-treated KK-Ay mice as control; KK-Ay+HUA, non-treated KK-Ay mice with hyperuricemia induced by combination of peritoneal injection of potassium oxonate at dose of 250mg/kg and intragastric administration of hypoxanthine at dose of 300mg/kg; KK-Ay+HUA+EMP, empagliflozin-treated KK-Ay mice with hyperuricemia induced by combination of peritoneal injection of potassium oxonate at dose of 250mg/kg and intragastric administration of hypoxanthine at dose of 300mg/kg. HE, hematoxylin-eosin staining; PAS, periodic acid-Schiff staining.

HUA is confirmed as an independent risk factor for kidney diseases 20-22, and chronic kidney disease (CKD) is a common comorbidity in T2DM 3, 23. By western blot, we observed SGLT2 was not only expressed in kidney, but also in ileum and the levels of ABCG2 in ileum were notably reduced in ileum compared with kidney (Supplemental Figure 2A-B). As ileum played an important role in UA excretion, we explored the mechanism not only in kidney but also in ileum. Hematoxylin-eosin (HE) and periodic acid-Schiff (PAS) staining showed that mice in the KK-Ay group and KK-Ay+HUA group displayed drastic tubular dilatation, hydropic degeneration and deposited mesangial matrix compared with mice in the C57BL/6J group, however, those pathological features were significantly attenuated in the KK-Ay+HUA+EMP group (Fig. 2A, C, Supplemental Figure 3A, C). We also examined the histological changes in ileum, and found empagliflozin remarkably improved the histology of ileum in the KK-Ay+HUA+EMP group (Fig. 2B, Supplemental Figure 3B).

### Empagliflozin promoted ABCG2 expression in kidney and ileum

**Figure 3:**
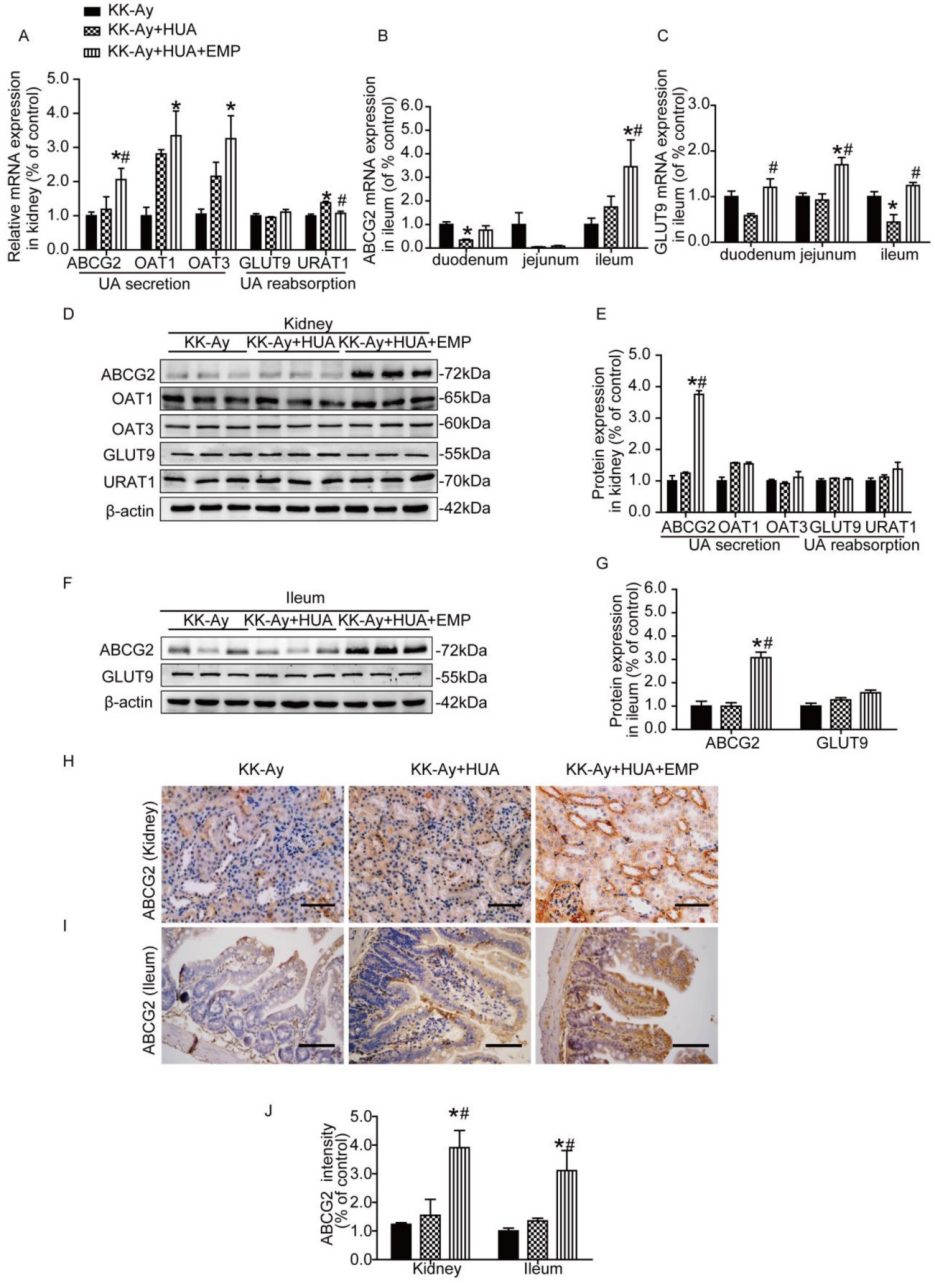
** Effects of empagliflozin on uric acid transporters in kidney and ileum from KK-Ay mice with hyperuricemia.** (A) Relative mRNA levels of ABCG2, OAT1, OAT3, GLUT9 and URAT1 in the kidney. Empagliflozin notably increased the mRNA levels of ABCG2 and significantly reduced the URAT1 mRNA compared with KK-Ay+HUA group in kidney, but had no effect on mRNA of OAT1, OAT3 and GLUT9 in kidney. (B-C) Relative mRNA levels of ABCG2 and GLUT9 in duodenum, jejunum and ileum. In ileum, empagliflozin remarkably up-regulated the mRNA levels of ABCG2. (D-E) Western blot and its analysis of ABCG2, OAT1, OAT3, GLUT9 and URAT1 protein expression in kidney. The protein levels of ABCG2, but not OAT1, OAT3, URAT1 or GLUT9 were obviously altered in the kidney after empagliflozin treatment. (F-G) Western blot and its analysis of ABCG2 and GLUT9 protein expression in ileum. Empagliflozin notably increased the protein levels of ABCG2, while did not significantly affect the expression of GLUT9 in ileum. (H-J) Immunochemistry staining of ABCG2 in kidney and ileum (original magnification × 400, bars = 100μm). ABCG2 levels were significantly increased in KK-Ay+HUA+EMP group. One-way ANOVA followed by Tukey's test was applied to compare the differences between any two of three groups. The data are presented as the mean ± SEM (n = 5 for each group). *P < 0.05 vs. KK-Ay group; ^#^P < 0.05 vs. KK-Ay+HUA group. KK-Ay, non-treated KK-Ay mice as control; KK-Ay+HUA, non-treated KK-Ay mice with hyperuricemia induced by combination of peritoneal injection of potassium oxonate at dose of 250mg/kg and intragastric administration of hypoxanthine at dose of 300mg/kg; KK-Ay+HUA+EMP, empagliflozin-treated KK-Ay mice with hyperuricemia induced by combination of peritoneal injection of potassium oxonate at dose of 250mg/kg and intragastric administration of hypoxanthine at dose of 300mg/kg.

To find the mechanism responsible for anti-hyperuricemic effect of empagliflozin, we evaluated the expression of the major UA transporters by RT-PCR, western blot and immunohistochemistry in both kidneys and small intestines. As shown in Fig. 3A and Supplemental figure 4A, empagliflozin notably increased the mRNA levels of ABCG2 and significantly reduced the URAT1 mRNA compared with KK-Ay+HUA group in kidney, but had no effect on mRNA of OAT1, OAT3 and GLUT9 in kidney. In small intestines, we observed that ABCG2 is mainly expressed in the ileum (Fig. 3B) and GLUT9 has similar expression trends in the three groups (Fig. 3C). In ileum, empagliflozin remarkably up-regulated the mRNA levels of ABCG2, but had no effect on GLUT9 mRNA expression (Fig. 3C, Supplemental figure 4B). Interestingly, results from western blot and immunohistochemistry demonstrated that the protein levels of ABCG2, but not OAT1, OAT3, URAT1 or GLUT9 were obviously altered in the kidneys after empagliflozin treatment (Fig. 3D-E, H-J, Supplemental Figure 4C-D, G, I, Supplemental Figure 5A-B). Similarly, in ileum, empagliflozin notably increased the protein levels of ABCG2, while did not significantly affect the expression of GLUT9 (Fig. 3F-G, I-J, Supplemental figure 4E-F, H-I).

### Empagliflozin promoted in AMPK/AKT/CREB pathway in kidney and ileum

**Figure 4:**
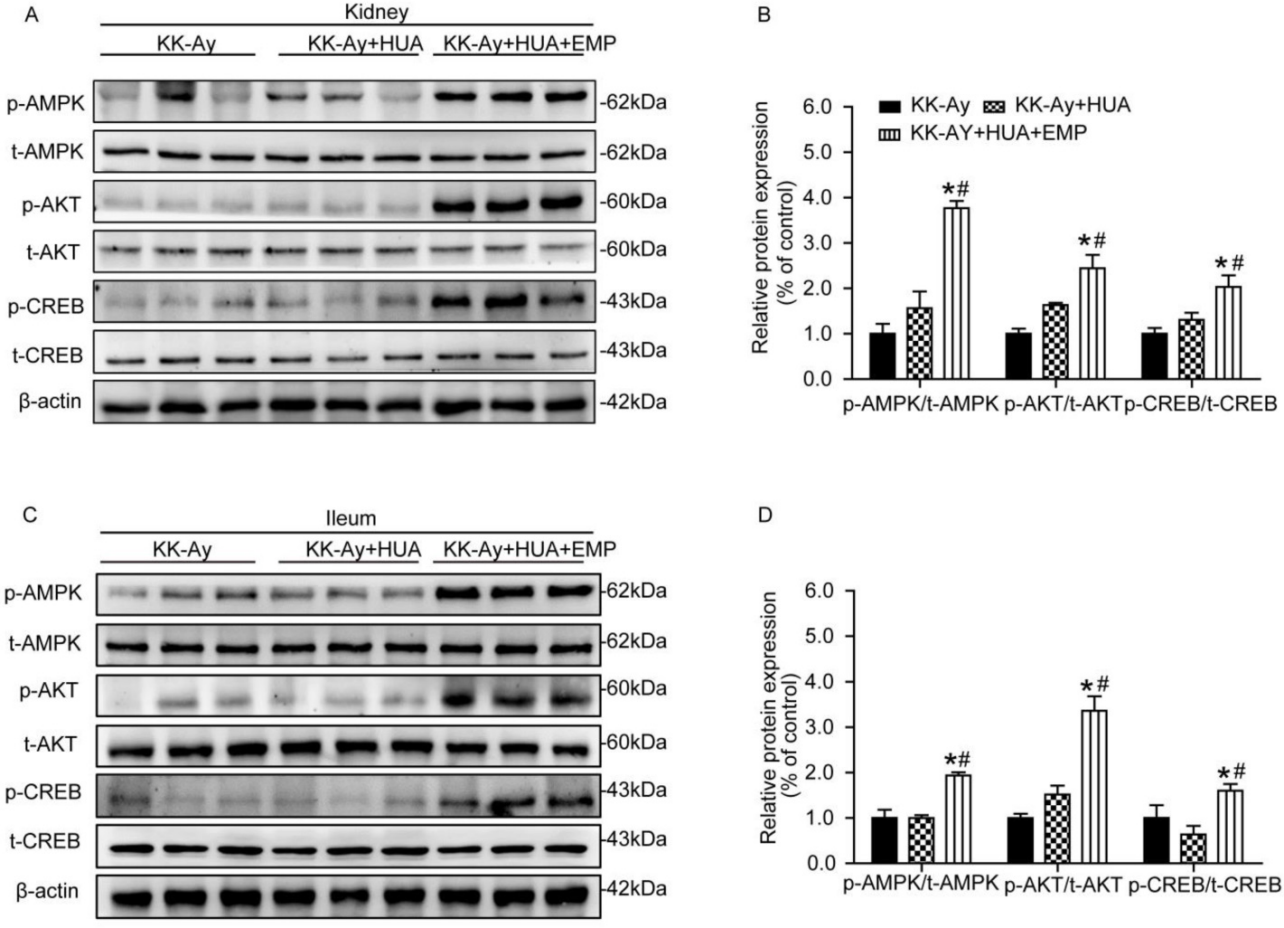
** Effects of empagliflozin on AMPK/AKT/CREB/ signaling in kidney and ileum from KK-Ay mice with hyperuricemia.** (A-B) Western blot and its analysis of p-AMPK, p-AKT and p-CREB in kidney. Levels of p-AMPK, p-AKT and p-CREB were obviously increased in KK-Ay+HUA+EMP group in kidney. (C-D) Western blot analysis of p-AMPK, p-AKT and p-CREB in ileum. Levels of p-AMPK, p-AKT and p-CREB were obviously increased in KK-Ay+HUA+EMP group in ileum. One-way ANOVA followed by Tukey's test was applied to compare the differences between any two of three groups. The data are presented as the mean ± SEM (n = 5 for each group). *P < 0.05 vs. KK-Ay group; ^#^P < 0.05 vs. KK-Ay+HUA group. KK-Ay, non-treated KK-Ay mice as control; KK-Ay+HUA, non-treated KK-Ay mice with hyperuricemia induced by combination of peritoneal injection of potassium oxonate at dose of 250mg/kg and intragastric administration of hypoxanthine at dose of 300mg/kg; KK-Ay+HUA+EMP, empagliflozin-treated KK-Ay mice with hyperuricemia induced by combination of peritoneal injection of potassium oxonate at dose of 250mg/kg and intragastric administration of hypoxanthine at dose of 300mg/kg.

As demonstrated in Fig. 4A-D, renal and ileal tissues were homogenized, and the levels of p-AMPK, p-AKT and p-CREB were examined. It turned out that no significant changes in the levels of p-AMPK, p-AKT and p-CREB were observed in kidney or ileum in mice from the KK-Ay group and KK-Ay+HUA group. In contrast, the levels of p-AMPK, p-AKT and p-CREB were sharply enhanced in the KK-Ay +HUA+EMP group.

### Empagliflozin promoted ABCG2 expression through AMPK/AKT/CREB pathway in HK-2 cells

**Figure 5:**
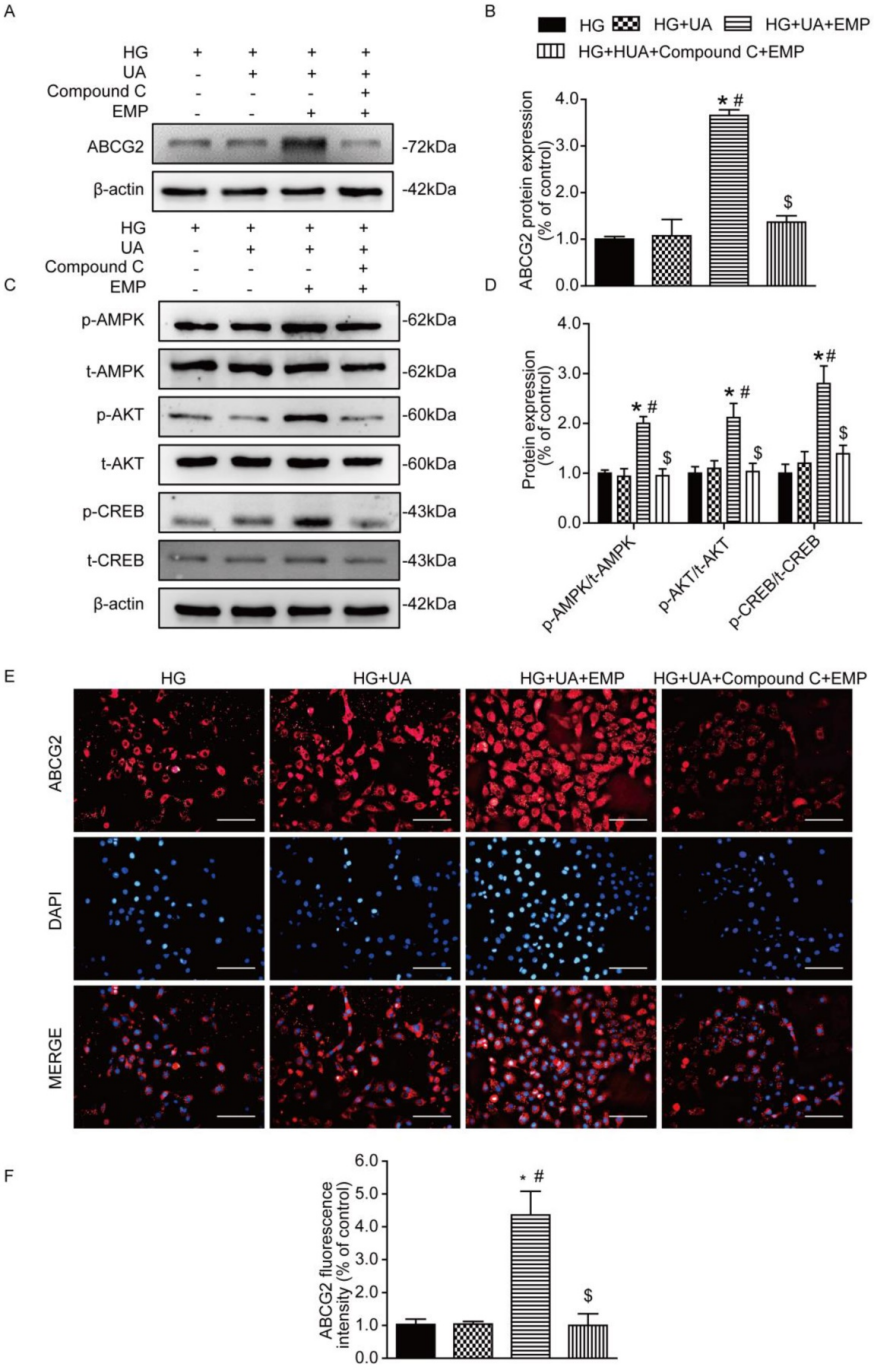
** Effects of empagliflozin on uric acid transporters and AMPK/Akt/CREB signaling in cultured HK-2 cells.** (A-B) Protein levels and analysis of ABCG2 were assessed by western blot. Levels of ABCG2 were significantly increased in HG+UA+EMP group compared with HG group and HG+UA group, while were decreased in HG+UA+ Compound C+EMP. (C-D) Phosphorylated levels and analysis of AMPK, AKT and CREB phosphorylation were assessed by western blot analysis. Empagliflozin significantly promoted phosphorylation of AMPK, AKT and CREB, which were blocked by Compound C. (E) Immunofluorescence staining of ABCG2 in HK-2 cells (original magnification × 100, bars = 10μm). Fluorescence intensity of ABCG2 was significantly increased in HG+UA+EMP group compared with HG group and HG+UA group, and was notably decreased in HG+UA+ Compound C+EMP. One-way ANOVA followed by Tukey's test was applied to compare the differences between any two of the four groups. The data are presented as the mean ± SEM (n = 3). *P < 0.05 vs. HG group; ^#^P < 0.05 vs. HG+UA group; ^$^P < 0.05 vs. HG+UA+EMP group. HG group, cells incubated in DMEM/HG medium; HG+UA group, cells incubated in UA (10mg/dl) added to DMEM/HG medium; HG+ HUA+EMP group, cells incubated in DMEM/HG medium with UA (10mg/dl) and treated with 50 μM empagliflozin; HG+UA+ Compound C group, cells incubated in DMEM/HG medium with UA (10mg/dl) , AMPK inhibitor Compound C (10μM) and empagliflozin (50 μM) for 48 hours.

To confirm the mechanism of empagliflozin, AMPK inhibitor (Compound C) was applied to cultured human tubular epithelial (HK-2) cells prior to empagliflozin treatment. Western blot and immunofluorescence showed that ABCG2 was significantly increased in the group of HG+UA+EMP. However, ABCG2 was notably decreased in the HG+UA +Compound C +EMP group (Fig. 5A-B, E-F). Moreover, we found that the levels of p-AMPK, p-AKT, p-CREB were obviously increased in the HG+UA+EMP group, while significantly decreased after treatment with Compound C (Fig. 5C-D). Our observation further suggested that empagliflozin promoted ABCG2 expression through AMPK/AKT/CREB pathway *in vitro*.

### CREB facilitated ABCG2 expression through promoter activation

**Figure 6:**
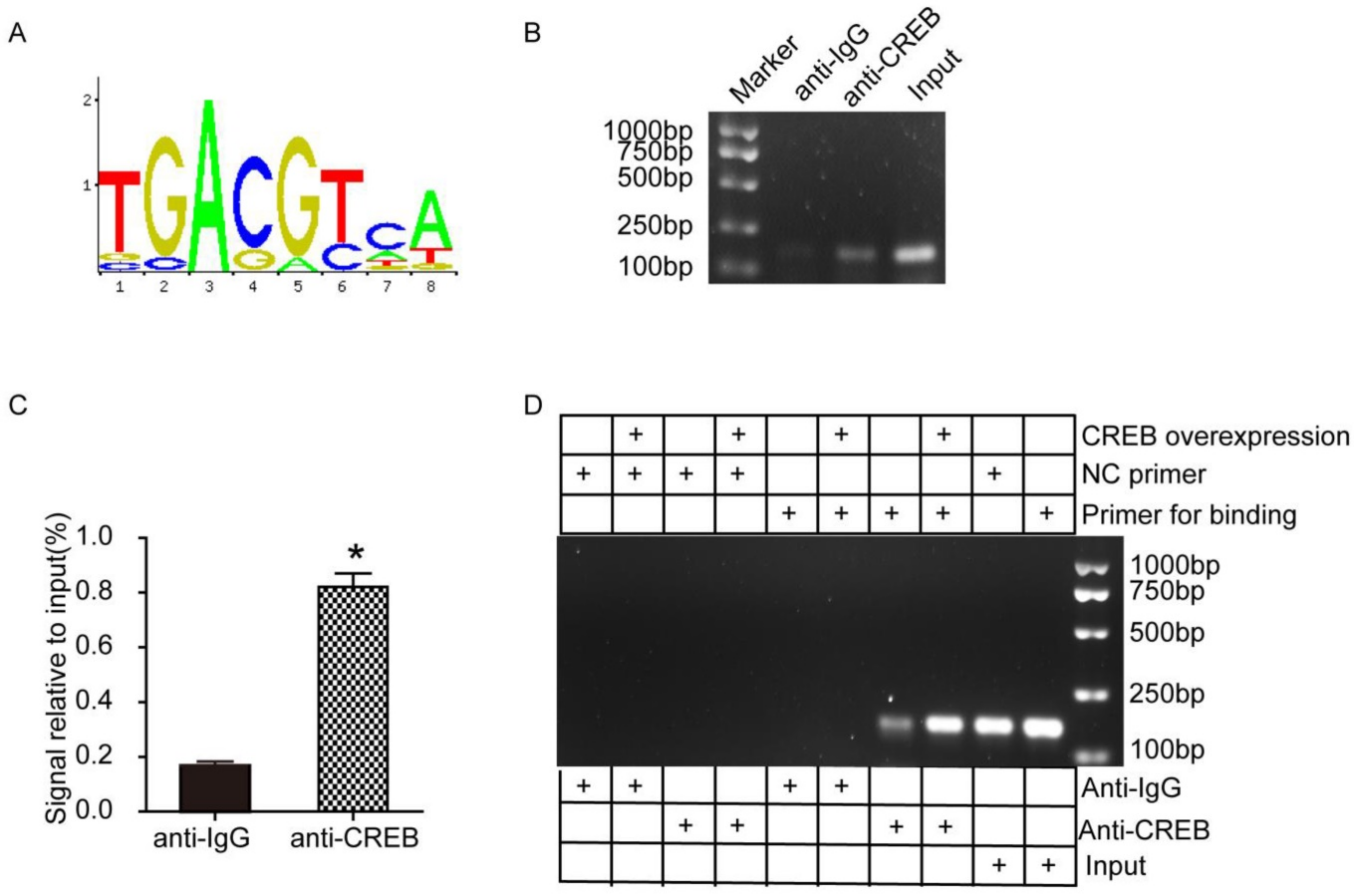
** CREB facilitated ABCG2 expression through promoter activation in HK-2 cells and HEK-293A cells.** (A) A consensus motif, a possibly predicted site sequence used for CREB binding to ABCG2 promoter. (B-C) ChIP assays showed the binding of CREB to ABCG2 promoter in HK-2 cells. (D) ChIP assays showed the binding of CREB to ABCG2 promoter in HEK-293A cells. Two tailed, unpaired Student's t-test was applied in statistical analysis between two groups. The data are presented as the mean ± SEM (n = 3). *P < 0.05 vs. anti-IgG group. CREB plasmid, cells transfected with CREB plasmid.

To further investigate the potential mechanism between CREB and ABCG2, we conducted ChIP assays using HK-2 cells. According to the JASPAR database, we predicted a motif that CREB facilitates ABCG2 expression (Fig. 6A). Then we designed 2 pairs of primers based on ABCG2 promoter region (Table. 1). As shown in Fig. 6B-C, ChIP-PCR results indicated that CREB may bind to ABCG2 promoter and the signal relative to input in anti-CREB group is dramatically higher than the negative anti-IgG group. We next examined whether CREB physically interacts with the ABCG2 promoter by ChIP experiments using HEK-293A cells transfected with CREB plasmid and results showed CREB activated ABCG2 via binding to the promoter of ABCG2 to induce transcription. (Fig. 6D).

### Luciferase reporters in HEK-293A cells after CREB binding to the ABCG2 promoter

**Figure 7:**
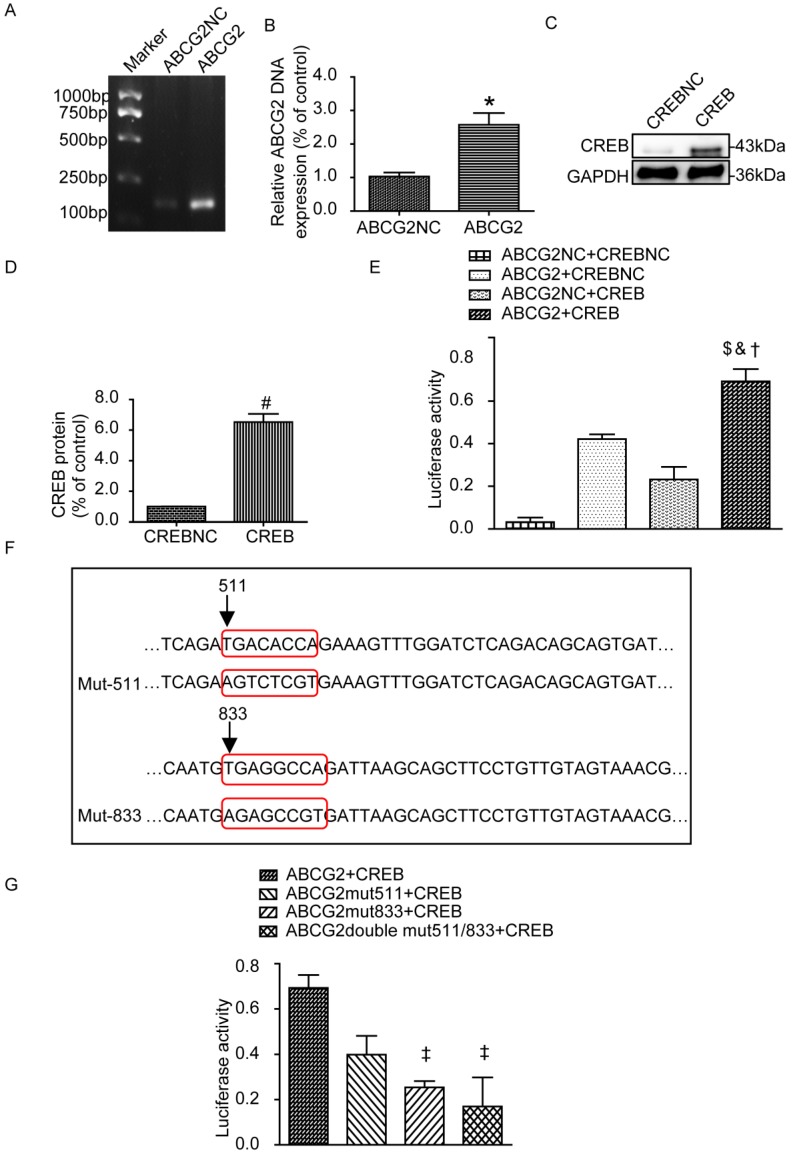
** Luciferase reporters in HEK-293A cells.** (A-B) Relative DNA levels of ABCG2. ABCG2 was overexpressed compared with their blank negative control. (C-D) Western blot and analysis of CREB in HEK-293A cells. CREB was overexpressed compared with their blank negative control. (E) Luciferase reporter gene assays of HEK-293A cells transiently co-transfected with different groups. The luciferase activity in the group of ABCG2+CREB was significantly increased. (F) Mutation sequence from 511 to 518, 833 to 840 sites or both of the ABCG2 promoter region. (G) Luciferase reporter gene assays of HEK-293A cells transiently co-transfected with different mutants. 833 to 840 mutant-sites group showed significantly reduced luciferase activity. Two tailed, unpaired Student's t-test was applied in statistical analysis between two groups. One-way ANOVA followed by Tukey's test was applied to compare the differences between any two of the four groups. The data are presented as the mean ± SEM (n = 3). *P < 0.05 vs. ABCG2NC group; ^#^P < 0.05 vs. CREBNC group; ^$^P < 0.05 vs. ABCG2NC+CREBNC group; ^&^P < 0.05 vs. ABCG2+CREBNC group; ^†^P < 0.05 vs. ABCG2NC+CREB group; ^‡^P < 0.05 vs. ABCG2+CREB group. ABCG2NC, empty plasmid as control for ABCG2 promoter plasmid; ABCG2, ABCG2 promoter plasmid; CREBNC, empty plasmid as control for CREB plasmid; CREB, CREB plasmid; ABCG2NC+CREBNC, empty plasmids as control for ABCG2 promoter plasmid and empty plasmid as control for CREB plasmid; ABCG2+CREBNC, ABCG2 promoter plasmid and empty plasmid as control for CREB plasmid; ABCG2NC+CREB, empty plasmid as control for promoter ABCG2 promoter plasmid and CREB plasmid; ABCG2+CREB, ABCG2 promoter plasmid and CREB plasmid; ABCG2 mut511+CREB, mutation of the promoter at 511 to 518 sites in ABCG2 promoter region and CREB plasmid; ABCG2 mut833+CREB, mutation of the promoter at 833 to 840 sites and CREB plasmid; ABCG2 double mut511/833+CREB, mutation of the promoter at 511 to 518 sites and 833 to 840 sites and CREB plasmid.

Next we examined the transfection efficiency of ABCG2 promoter plasmid and CREB plasmid by PCR and western blot, respectively. As shown in Fig. 7A-D, ABCG2 and CREB were overexpressed compared with their blank negative control. We also assessed whether CREB binding to the ABCG2 promoter by Gaussia luciferase reporter assays. HEK-293A cells were next transiently co-transfected with the following four groups: ABCG2NC+CREBNC, ABCG2+CREBNC, ABCG2NC+CREB, ABCG2+CREB. As expected, the luciferase activity in the group of ABCG2+CREB was significantly increased (Fig. 7E). The plasmid maps can be obtained in the Supplemental figure 6. Then, we mutated the 511 to 518 sites, 833 to 840 sites or both of them in the ABCG2 promoter region and co-transfected with CREB plasmid respectively. Results showed that 833 to 840 mutant-sites group showed significantly reduced luciferase activity but it was not obvious for 511 to 518 mutant-sites group (Fig. 7F-G).

## Discussion

The present study was firstly conducted in the model of T2DM mice with HUA, and demonstrated that empagliflozin treatment improved HUA through enhancing ABCG2 expression via AMPK/AKT/CREB pathway in both kidney and ileum. A remarkable improvement in biochemical parameters and histopathologic changes *in vivo* were observed after empagliflozin treatment, indicating the SUA-lowering effect of empagliflozin. Notably, our data from HK-2 cells after empagliflozin and AMPK inhibitor Compound C treatment further confirmed the mechanism that empagliflozin exerted anti-hyperglycemic effect *in vitro*.

HUA carries a significant risk for developing various diseases in T2DM 4-8, therefore, exploring the metabolism of UA and associated diseases has attracted researchers' much attention. However, it is a challenge to create hyperuricemic models in mice, because in rodents, uricase oxidizes UA to allantoin which is highly soluble in water and excreted unchanged in the urine, but not in humans 24. As potassium oxonate (PO) can block uricase, and hypoxanthine (HX) is the precursor of UA which is finally oxidized into UA by xanthine oxidase, combination of PO and HX has been widely used to induce HUA in rodents 25-27. As our data indicated, we successfully induced HUA in KK-Ay mice by combination of peritoneal injection of PO and intragastric administration of HX. Meanwhile, empagliflozin administration markedly suppressed SUA in KK-Ay mice with HUA, suggesting the hypouricemic effect of empagliflozin in T2DM mice.

It is well known that kidney is the main organ for excretion of approximately two thirds of the total UA 28. UA handling involves several classes of UA transporters responsible for UA handing. In brief, after glomerular filtration, UA is reabsorbed through URAT1 and GLUT9, and secreted through ABCG2, OAT1, and OAT3. According to previous studies, about one third of the total UA was excreted into the intestinal tract 29, and decreased extra-renal UA excretion is a common cause of HUA 30. Intestinal transporters play significant roles in mediating UA mobilization as well. ABCG2 is expressed on the apical membrane in intestines as well as kidneys 31. ABCG2 regulates SUA via physiologically important roles in both renal and intestinal UA excretion 31. Increased SUA in patients with ABCG2 dysfunction could be explained by the decreased excretion of UA from the intestine 32. Previous studies suggested that GLUT9 which was localized to the apical and basolateral enterocyte membranes, was abundantly expressed in intestine 33, 34, especially in the jejunum and ileum 34. Furthermore, it functioned to regulate enterocyte UA clearance, and enterocyte GLUT9-deficient mice developed HUA, hyperuricosuria and early-onset metabolic syndrome 34. Consistently, we detected low expression of ABCG2 in the KK-Ay group and KK-Ay+HUA group. In contrast, after empagliflozin administration, the SUA were decreased and the ABCG2 expression was up-regulated in both renal and ileal tissues in KK-Ay mice. This finding suggested that empagliflozin might exert anti-hyperuricemic effects by facilitating UA excretion through promoting ABCG2 expression in kidneys and ilea.

SGLT2 inhibitors have recently emerged as a promising novel class of glucose lowering drugs to facilitate glucose excretion in urine independent of insulin 35. Accumulating data indicated their anti- hyperuricemic effects in both clinical 18, 19, 36 and basic studies 37, 38. One of basic studies performed *in vitro* using Xenopus oocytes indicated that luseogliflozin lowered the SUA level due to inhibition of UA reabsorption mediated by GLUT9 isoform 2 at the collecting duct of the renal tubule 38. Another study conducted in STZ-induced diabetic rats suggested that empagliflozin may exert anti-hyperuricemic effects via regulating URAT1 and ABCG2 37. However, our study demonstrated that empagliflozin promoted ABCG2 expression in kidney and ileum in KK-Ay mice with HUA, but has little impact on the other transporters. The difference of the animal model may explain the difference to a certain extent. Although SGLT2 transporters are primarily located in the kidney, they are also found in other tissues, such as the small intestine, especially ileum 39. In our investigation, we also confirmed that SGLT2 was expressed in ileum (Supplemental figure. 2A-B).

Empagliflozin has been previously reported to trigger AMPK activation 40, which increased phosphorylation of AKT 41, resulting in CREB phosphorylation 42. Furthermore, a previous study implied that the CREB facilitated ABCG2 expression through promoter activation 43. The present study showed that empagliflozin treatment significantly promoted CREB directly bind to ABCG2 promoter through activating AMPK/AKT/CREB pathway. Moreover, with the application of AMPK inhibitor (Compound C), our data further confirmed that empagliflozin enhanced ABCG2 expression by promoting phosphorylation of AMPK, AKT and CREB.

## Conclusions

**Figure 8:**
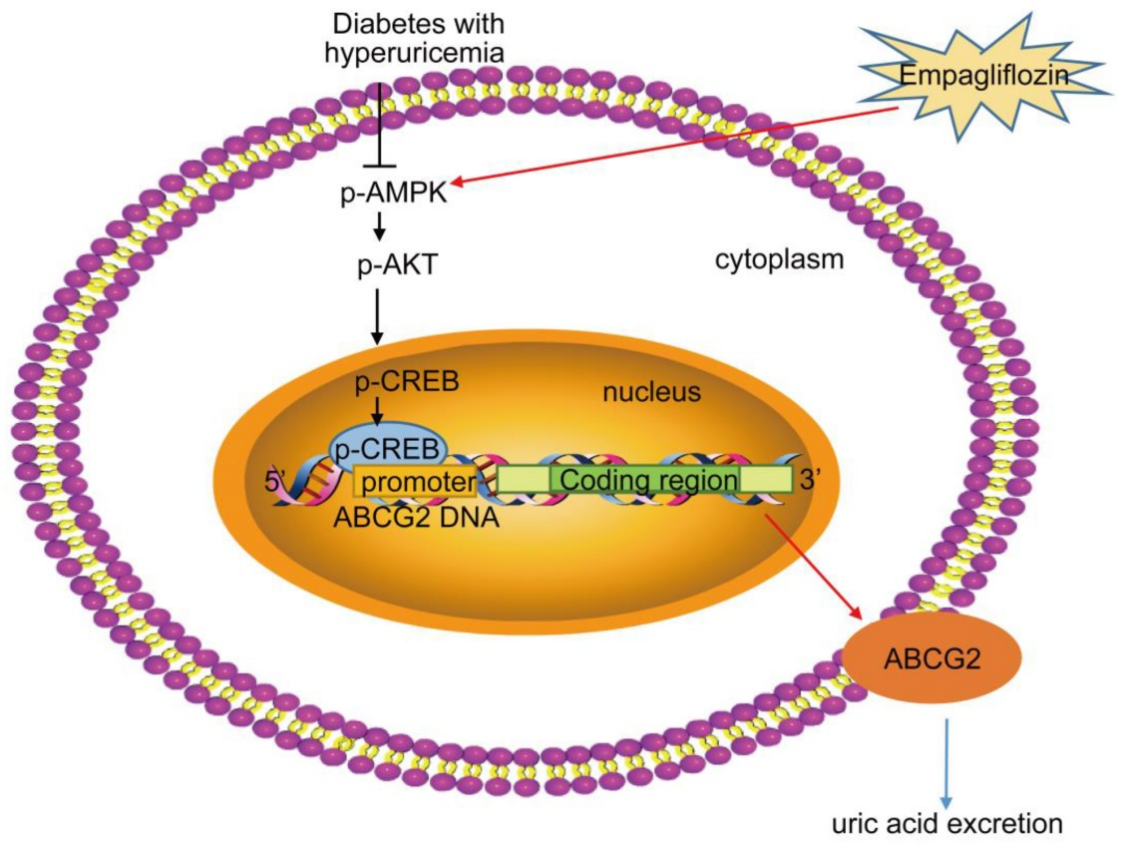
** Proposed mechanism of UA reduction by empagliflozin.** Empagliflozin treatment improved hyperuricemia by promoting UA excretion through up-regulating ABCG2 expression in diabetes with hyperuricemia. Furthermore, empagliflozin treatment promoted the phosphorylation of AMPK, AKT and CREB and further activated ABCG2 by facilitating CREB binding to the promoter of ABCG2 to induce transcription.

As summarized in Figure 8 our study demonstrated that empagliflozin treatment possessed anti-hyperglycemic effects and was attributed to UA excretion promotion through up-regulating ABCG2 expression in kidney and ileum in KK-Ay mice with HUA and in HK-2 cells. We also found that empagliflozin promoted the phosphorylation of AMPK, AKT and CREB and further activated ABCG2 by facilitating CREB binding to the promoter of ABCG2 to induce transcription. The findings in our studies lead to a new understanding of the mechanism about the effects of empagliflozin on improving HUA, and provided novel insights into the targeted therapies for HUA. Further studies are needed to explore whether other mechanisms are also involved in the SUA-lowering effect of empagliflozin in T2DM with HUA.

## Materials and Methods

### Animals

Six-week-old male C57BL/6J (24±0.5g) and KK-Ay mice (25 ± 0.4g) were obtained from Beijing HFK Bioscience Co. Ltd. (Beijing, China). They were housed at 24 ± 2°C. C57BL/6J mice were fed regular chow and there were considered as control group. KK-Ay mice were a kind of T2DM mouse model, and allowed free access to high-fat diet which consisted of 48.5% carbohydrates, 17.5% protein, 17.9% fat (Beijing HFK Bio-Technology Co. Ltd). KK-Ay mice were randomly assigned to 3 groups: diabetic control group (KK-Ay group, n=10), T2DM with HUA group (KK-Ay+HUA group, n=10), empagliflozin-treated KK-Ay mice with HUA group (KK-Ay+HUA+EMP group, n=10). The hyperuricemic model was induced by combination of peritoneal injection of PO at dose of 250mg/kg and intragastric administration of HX at dose of 300mg/kg daily, and mice in the KK-Ay+HUA+EMP group were orally administered with empagliflozin (10 mg/kg) in KK-Ay mice with HUA daily for 8 weeks. The dose of empagliflozin was determined based on previous studies 44. The study was approved by the ethical committee of Tianjin Medical University, and all procedures involving mice were conducted according to the Guide for the Care and Use of Laboratory Animals of the National Institutes of Health as well as the guidelines of the Animal Welfare Act.

### Biochemical Analysis

At the end of treatment, 10 mice from each group were placed in individual metabolic cages for 24 hours to collect the urine. The samples of blood were obtained from the retroorbital venous plexus. The blood and the urine were then used for analysis of SUA, urine UA with an automatic biochemistry analyzer (Roche). The fractional excretion of uric acid (FEUA) was calculated according to the formula: FEUA= (urine UA× serum creatinine) / (serum UA × urine creatinine) × 100, expressed as percentage 45. Mice were sacrificed by intraperitoneal injection of chloral hydrate.

### Hematoxylin-Eosin Staining and Periodic Acid-Schiff Staining of Renal Tissues

Kidneys were fixed in 4% paraformaldehyde and were then made into paraffin blocks. After deparaffinization and dehydration, HE staining and PAS staining of renal tissues were performed according to the manufacturer's instructions (Leagene Biotechnology, Beijing).

### Immunohistochemistry

For Immunohistochemistry staining, kidney sections were heated in sodium citrate buffer (pH 6.0) for antigen retrieval. After they were blocked with 3% H_2_O_2_ and 5% BSA, they were incubated with primary antibodies ABCG2 (1:200, Affinity), GLUT9 (1:100, Novus), URAT1 (1:100, Abbiotec), OAT1 (1:100, Bioworld), OAT3 (1:100, Bioworld) overnight at 4 °C. Secondary antibodies were subsequently used to incubate for 30 minutes at 37°C and a diaminobenzidine (DAB) kit was performed to visualize the positive expression. After that, sections counterstained with haematoxylin. Stained sections were captured with a light microscopy in 10 randomly cortical sections (×400) and quantified with Image Pro Plus 6.0 software.

### Cell culture

HK-2 cells were cultured with DMEM/F12 medium containing 10% fetal bovine serum (FBS) and 1% penicillin/streptomycin in the presence of 5% CO2. The cells were divided into four groups: (1) high glucose (HG) group incubated in DMEM/HG medium, (2) HG+UA group incubated in UA (10mg/dl) added to DMEM/HG medium (HG+ UA) group, (3) HG+UA+EMP group incubated in DMEM/HG medium with UA (10mg/dl) and treated with 50 μM empagliflozin, (4) HG+UA+ Compound C group incubated in DMEM/HG medium with UA (10mg/dl), AMPK inhibitor Compound C (10μM) and empagliflozin (50 μM) for 48 hours.

### Transfection

HEK-293A cells were cultured with DMEM/HG medium containing 10% FBS and 1% penicillin/ streptomycin. Transient transfection was performed using Lipofectamine 2000 (Invitrogen, USA) according to the manufacturer's instructions. To study whether CREB activated ABCG2 expression by facilitating CREB binding to the promoter of ABCG2, HEK-293A cells were divided to the following groups: (1) empty plasmid as control for ABCG2 promoter plasmid (ABCG2NC), (2) ABCG2 promoter plasmid (ABCG2), (3) empty plasmid as control for CREB plasmid (CREBNC), (4) CREB plasmid (CREB), (5) ABCG2 promoter plasmid and empty plasmid as control for CREB plasmid (ABCG2+CREBNC), (7) empty plasmid as control for ABCG2 promoter plasmid and CREB plasmid (ABCG2NC+CREB), (8) ABCG2+CREB, ABCG2 promoter plasmid and CREB plasmid, (9) mutation of the promoter at 511 to 518 sites in ABCG2 promoter region and CREB plasmid (ABCG2mut511+CREB); (10) mutation of the promoter at 833 to 840 sites and CREB plasmid (ABCG2mut833+CREB); (11) mutation of the promoter at 511 to 518 sites and 833 to 840 sites and CREB plasmid (ABCG2 double mut511/833+CREB).

### Quantitative RT-PCR

Total RNA was extracted using E.Z.N.A. Total RNA Kits (OMEGA, GA, USA). 1 μg of RNA was reverse-transcribed with Revert Aid First Strand cDNA Synthesis Kits (Thermo, MA, USA) according to the manufacturer's instructions. Reverse transcription polymerase chain reaction (RT-PCR) was performed using the CFX96 real-time PCR system (Bio-Rad, USA) with the SYBR Green PCR Kit (Takara, Otsu, Japan). The primers were listed in the table 2.

### Western blot analysis

After protein extracts from kidney, ileum and cells were boiled in lysis buffer, and they were subsequently loaded in 10% SDS-PAGE gels for electrophoresis. Then the protein was transferred to a PVDF membrane (Millipore, MA, USA). After blocked in 5% non-fat milk in TBST for 2 hours at room temperature, the membrane was incubated with primary antibody overnight at 4 °C. The primary antibodies included ABCG2 (1:100, Santa Cruz Biotechnology), GLUT9 (1:1000, Novus), URAT1 (1:200, Abbiotec), OAT1 (1:500, Bioworld), OAT3 (1:500, Bioworld), p-AMPK (1:1000, Cell Signaling), AMPK (1:1000, Cell Signaling), p-CREB (1:1000, Cell Signaling), CREB (1:1000, Cell Signaling), p-AKT (1:1000, Cell Signaling), AKT (1:1000, Cell Signaling). After that, they were incubated with the appropriate secondary antibody for 2 hours at room temperature. ECL kit (Advansta, USA) was used to visualize the bands and ImageJ software was performed to quantify the band intensity.

### Immunofluorescence staining

After 48 hours of intervention, different groups of HK-2 cells cultured in 24-well plates were fixed with 4% paraformaldehyde. After that, cells were blocked with 1% BSA and then incubated with anti-ABCG2 (1:25, Affinity) at 4°C overnight. Then, the cells were incubated with secondary antibodies at 37 °C for 1 hour and cell nucleus was stained by DAPI for 2 minus in the dark.

### Chromatin immunoprecipitation assay

Cells were first cross-linked with 1% formaldehyde, and then lysed by Micrococcal Nuclease (Cell Signaling). Chromatin was harvested and subjected to immunoprecipitation with IgG antibody and p-CREB antibody (1:50, Cell Signaling). The protein-DNA complexes were reversed and the DNA was purified using the Simple ChIP Kit (Cell Signaling). The enrichment of the particular DNA sequence was detected by PCR (Bio-Rad, USA).

### Luciferase reporter gene assay

A luciferase reporter gene assay was performed using a Secrete-Pair Gaussia Luciferase Assay kit (Gene Copoeia, Inc.). HEK-293A cells were seeded at a density of 1.5x10^4^/ml into 96-well plates. After 12 hours, the cells were transiently co-transfected with 0.1 μg plasmid per well. 48 hours after transfection, supernatants of transfected cells were collected, and then the luciferase activity in the supernatants was measured using SynergyMx (BioTek, USA) according to the manufacturer's instructions.

### Statistical Analysis

All the data were presented as mean ± standard error of mean (SEM). Two tailed, unpaired Student's t-test was applied in statistical analysis between two groups. One-way analysis of variance (ANOVA) was performed to determine statistical significance among more than three groups, and Tukey's post hoc test was used to compare any two groups among them. P < 0.05 was considered significant.

## Supplementary Material

Supplementary figures.

## Figures and Tables

**Table 1:** Primer sequence used in real time RT-PCR

Site		Sequence (5' to 3')
511	Forward	AGGAAGCCAGGAAGACAT
	Reverse	CTTCAGGCTCCTAATGGT
833	Forward	ACGGTATGATTGATTCTTC
	Reverse	AAGGTCGTTTACTACAACAG

**Table 2:** Primer sequence used in real time RT-PCR

Gene (protein)		Sequence (5' to 3')
ABCG2	Forward	CAGCTGTGGAGCTGTTCGTA
	Reverse	GGCAAGAACCTCATGGGGAG
SLC22A6 (OAT1)	Forward	CTAGGGAAAGGCTGTCTGGC
	Reverse	AGCGCCGAAGATGAAGAGAG
SLC22A8 (OAT3)	Forward	CCTGGCCCTCATCTTTGTGT
	Reverse	CATACTTCCCACTCGAGCCC
SLC2A9 (GLUT9)	Forward	GGGTGTTCCTGGCTACCTTC
	Reverse	CAGGTGTAGTGCTGGGTCAG
SLC22A12 (URAT1)	Forward	CGATGTTCTTCTGGCCGTCT
	Reverse	TGGTCGTAAACCCAGCCATC
GAPDH	Forward	GGAGAGTGTTTCCTCGTCCC
	Reverse	ATGAAGGGGTCGTTGATGGC

## Abbreviations

ABCG2: ATP-binding cassette subfamily G member 2
AMPK: AMP-activated protein kinase
ANOVA: one-way analysis of variance
CKD: chronic kidney disease
CREB: cAMP-response element binding protein
DAB: diaminobenzidine
FBS: fetal bovine serum
GLUT9: glucose transporter 9
HE: hematoxylin-eosin
HG: high glucose
HK-2: human kidney proximal tubular epithelial cells
HUA: hyperuricemia
HX: hypoxanthine
OAT1: organic anion transporter 1
OAT3: organic anion transporter 3
PAS: periodic acid-Schiff
PO: potassium oxonate
RT-PCR: reverse transcription polymerase chain reaction
SEM: standard error of mean
SGLT2: sodium-glucose co-transporter 2
SUA: serum uric acid
T2DM: type 2 diabetes
UA: uric acid
URAT1: urate transporter 1
