# Supplementary material for: Empagliflozin Attenuates Hyperuricemia by Upregulation of ABCG2 via AMPK/AKT/CREB Signaling Pathway in Type 2 Diabetic Mice
Source: Int J Biol Sci. 2020 Jan 1;16(3):529–42. doi: 10.7150/ijbs.33007 (PMC6990905; doi:10.7150/ijbs.33007)
Supplement: Supplementary file 1 — Supplementary figures. [file ijbsv16p0529s1.pdf]

Figure Legends

Supplemental Figure 1. Urinary excretion of uric acid from C57BL/6J mice, KK-Ay mice, KK-Ay mice with hyperuricemia, empagliflozin-treated KK-Ay mice with hyperuricemia. (A-G) Body weight, blood glucose, serum uric acid, urinary uric acid and FEUA, urinary glucose excretion, urine volume in the C57BL/6J group, KK-Ay group, KK-Ay+HUA group and KK-Ay +HUA+EMP group after 8 week-treatment with empagliflozin. The body weight, blood glucose, urinary glucose excretion, urine volume of KK-Ay mice, KK-Ay mice with hyperuricemia, empagliflozin-treated KK-Ay mice with hyperuricemia were obviously increased than that of C57BL/6J mice, and the urinary uric acid, FEUA of the three groups were obviously decreased than the C57BL/6J group. One-way ANOVA followed by Tukey's test was applied to compare the differences between any two of the four groups. The data are presented as the mean ± SEM (n = 5 for each group). $^{☆}P < 0.05$ vs. C57BL/6J group; $*P < 0.05$ vs. KK-Ay group; $^{\#}P < 0.05$ vs. KK-Ay+HUA group. C57BL/6J, non-treated C57BL/6J mice as control; KK-Ay, non-treated KK-Ay mice; KK-Ay+HUA, non-treated KK-Ay mice with hyperuricemia induced by combination of peritoneal injection of potassium oxonate at dose of 250mg/kg and intragastric administration of hypoxanthine at dose of 300mg/kg; KK-Ay+HUA+EMP, empagliflozin-treated KK-Ay mice with hyperuricemia induced by combination of peritoneal injection of potassium oxonate at dose of 250mg/kg and intragastric administration of hypoxanthine at dose of 300mg/kg. FEUA, fractional excretion of uric acid.

Supplemental Figure 2. SGLT2 protein expression was expressed in both kidney and ileum. (A-B) Western blot and analysis of SGLT2 protein expression in kidney and ileum. SGLT2 was expressed in both kidney and ileum and the levels of SGLT2 in ileum were notably reduced compared with kidney. $*P < 0.05$ vs. kidney group. Two tailed, unpaired Student's t-test was applied in statistical analysis between two groups.

Supplemental Figure 3. Histopathologic changes of kidney and ileum from C57BL/6J mice, KK-Ay mice, KK-Ay mice with hyperuricemia, empagliflozin-treated KK-Ay mice with hyperuricemia. (A) Photomicrographs of kidney (original magnification × 400, bars = 50μm). C57BL/6J mice showed normal structure of kidney. Red arrows indicate the tubular dilatation, blue arrows indicate the hydropic degeneration and white arrows indicate deposited mesangial matrix. (B) Photomicrographs of ileum (original magnification × 400, bars = 50μm). C57BL/6J mice presented normal structure of ileum. Yellow arrows indicate abnormality of ileum. (C) Analysis of mesangial area expansion. One-way ANOVA followed by Tukey's test was applied to compare the differences between any two of the four groups. The data are presented as the mean ± SEM (n = 5 for each group). $^{☆}P < 0.05$ vs. C57BL/6J group; $^{*}P < 0.05$ vs. KK-Ay group; $^{\#}P < 0.05$ vs. KK-Ay+HUA group. C57BL/6J, non-treated C57BL/6J mice as control; KK-Ay, non-treated KK-Ay mice as control; KK-Ay+HUA, non-treated KK-Ay mice with hyperuricemia induced by combination of peritoneal injection of potassium oxonate at dose of 250mg/kg and intragastric administration of hypoxanthine at dose of 300mg/kg; KK-Ay+HUA+EMP, empagliflozin-treated KK-Ay mice with hyperuricemia induced by combination of peritoneal injection of potassium oxonate at dose of 250mg/kg and intragastric administration of hypoxanthine at dose of 300mg/kg. HE, hematoxylin-eosin staining; PAS, periodic acid–Schiff staining.

Supplemental Figure 4. Uric acid transporters in kidney and ileum from C57BL/6J mice, KK-Ay mice, KK-Ay mice with hyperuricemia, empagliflozin-treated KK-Ay mice with hyperuricemia. (A) Relative mRNA levels of ABCG2, OAT1, OAT3, GLUT9 and URAT1 in kidney. Relative mRNA levels of ABCG2, OAT1, OAT3, URAT1 from KK-Ay group, KK-Ay+HUA group, KK-Ay+HUA+EMP group were obviously decreased compared with the C57BL/6J mice, while there was no significant difference in GLUT9 mRNA levels among the four group. (B) Relative mRNA levels of ABCG2 and

GLUT9 in ileum. The mRNA levels of ABCG2 in KK-Ay group, KK-Ay+HUA group, KK-Ay+HUA+EMP group were significant downregulated compared with the C57BL/6J mice in ileum. (C-D) Western blot and its analysis of ABCG2, OAT1, OAT3, GLUT9 and URAT1 protein expression in kidney. The ABCG2 protein levels in kidney from KK-Ay group, KK-Ay+HUA group were notably decreased compared with C57BL/6J group, while the ABCG2 protein levels in kidney between C57BL/6J group and KK-Ay+HUA+EMP group showed no significantly different. (E-F) Western blot and its analysis of ABCG2 and GLUT9 protein expression in ileum. The ABCG2 protein levels in ileum from KK-Ay group, KK-Ay+HUA group showed obviously downregulated compared with C57BL/6J group, while we observed no obvious difference in ABCG2 protein levels between C57BL/6J group and KK-Ay+HUA+EMP group. (G-I) Immunochemistry staining of ABCG2 in kidney and ileum (original magnification × 400, bars = 100μm). The four groups presented similar trend as the western blot. One-way ANOVA followed by Tukey's test was applied to compare the differences between any two of the four groups. The data are presented as the mean ± SEM (n = 5 for each group). ☆P < 0.05 vs. C57BL/6J group; *P < 0.05 vs. KK-Ay group; #P < 0.05 vs. KK-Ay+HUA group. C57BL/6J, non-treated C57BL/6J mice as control; KK-Ay, non-treated KK-Ay mice as control; KK-Ay+HUA, non-treated KK-Ay mice with hyperuricemia induced by combination of peritoneal injection of potassium oxonate at dose of 250mg/kg and intragastric administration of hypoxanthine at dose of 300mg/kg; KK-Ay+HUA+EMP, empagliflozin-treated KK-Ay mice with hyperuricemia induced by combination of peritoneal injection of potassium oxonate at dose of 250mg/kg and intragastric administration of hypoxanthine at dose of 300mg/kg.

Supplemental Figure 5. Uric acid transporters in kidney from C57BL/6J mice, KK-Ay mice, KK-Ay mice with hyperuricemia, empagliflozin-treated KK-Ay mice with hyperuricemia. (A) Immunochemistry staining of URAT1, GLUT9, OAT1 and OAT3 in kidney (original magnification × 400, bars = 50μm). There was no significant difference in uric acid transporters among the four groups. One-way ANOVA followed by Tukey's test was applied to compare the differences between any two of the four groups. The data are presented as the mean ± SEM (n = 5 for each group). ☆P < 0.05 vs. C57BL/6J group; *P < 0.05 vs. KK-Ay group; #P < 0.05 vs. KK-Ay+HUA group. C57BL/6J, non-treated C57BL/6J mice as control; KK-Ay, non-treated KK-Ay mice as control; KK-Ay+HUA, non-treated KK-Ay mice with hyperuricemia induced by combination of peritoneal injection of potassium oxonate at dose of 250mg/kg and intragastric administration of hypoxanthine at dose of 300mg/kg; KK-Ay+HUA+EMP, empagliflozin-treated KK-Ay mice with hyperuricemia induced by combination of peritoneal injection of potassium oxonate at dose of 250mg/kg and intragastric administration of hypoxanthine at dose of 300mg/kg.

Supplemental Figure 6. Plasmids map. (A) Plasmids maps of ABCG2NC, ABCG2, CREBNC, CREB. ABCG2NC, empty vector as control for ABCG2 promoter plasmid; ABCG2, ABCG2 promoter plasmid; CREBNC, empty vector as control for CREB plasmid; CREB, CREB plasmid.

Supplemental figure 1

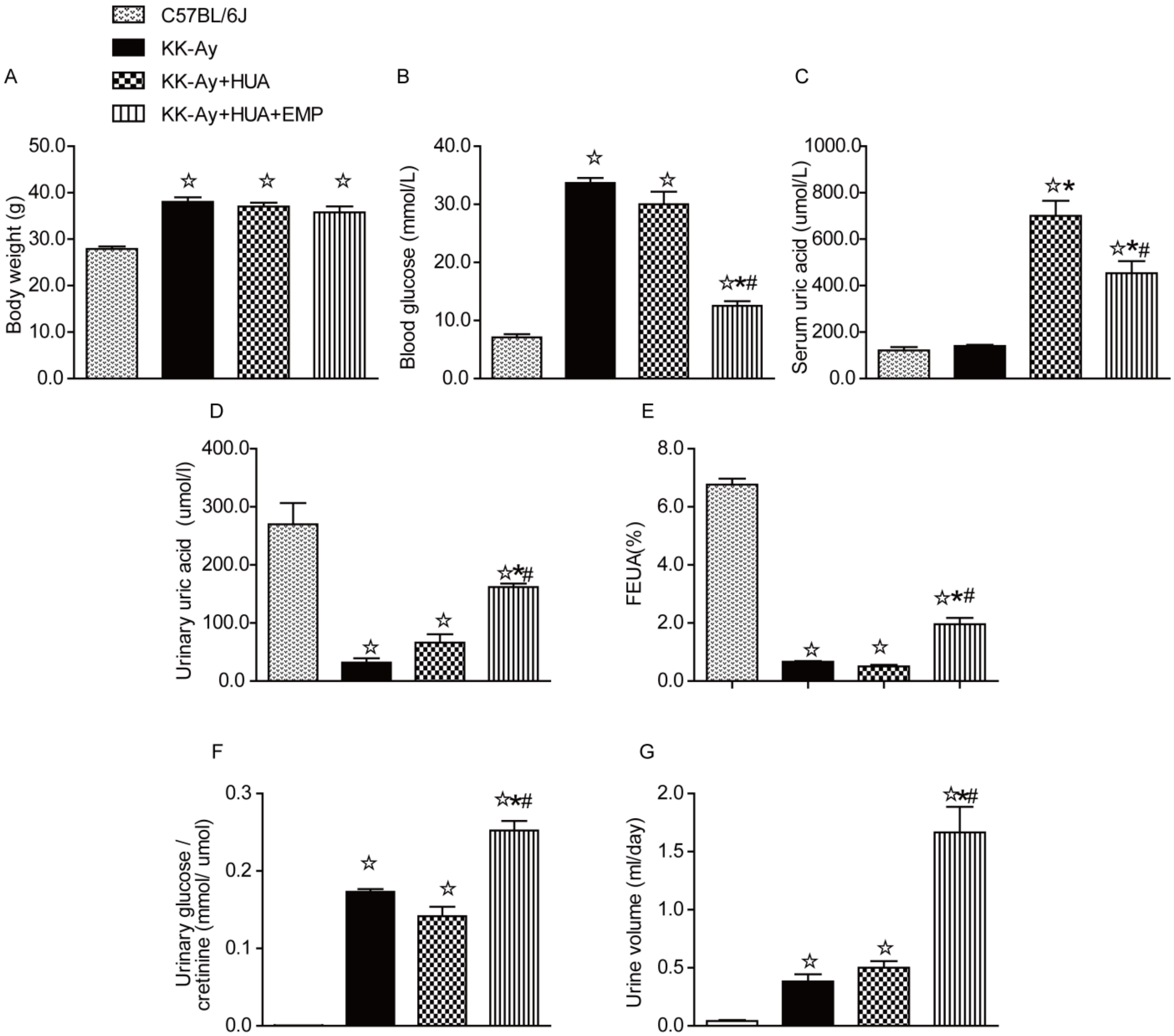

Supplemental figure 2

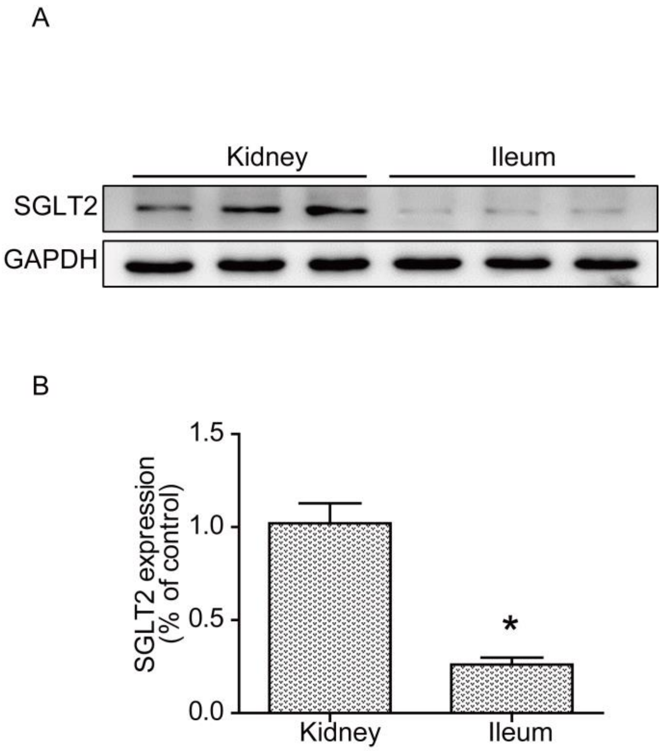

Supplemental figure 3

A

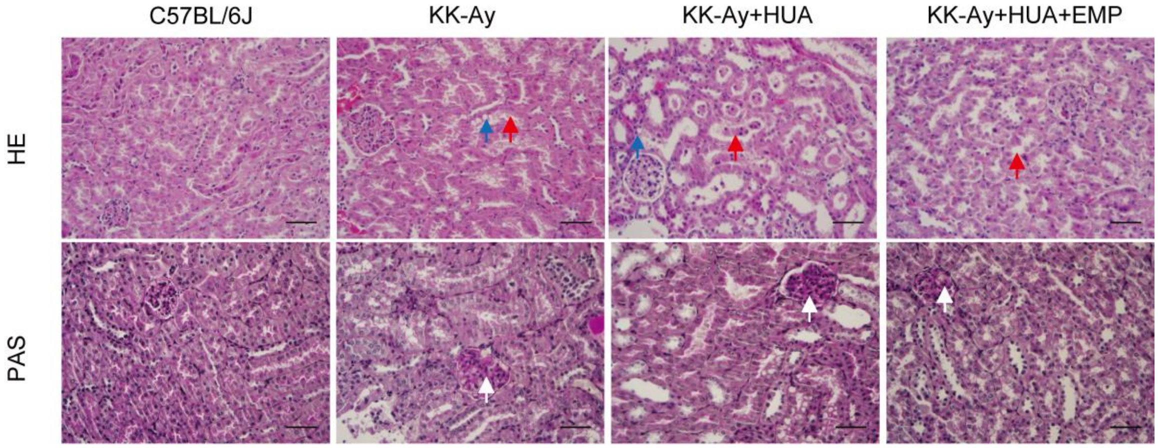

B

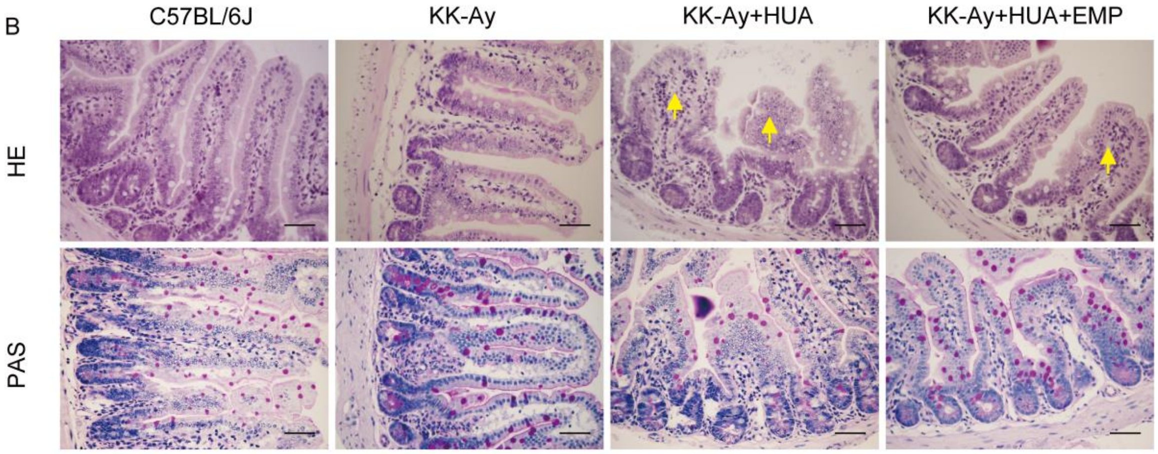

C

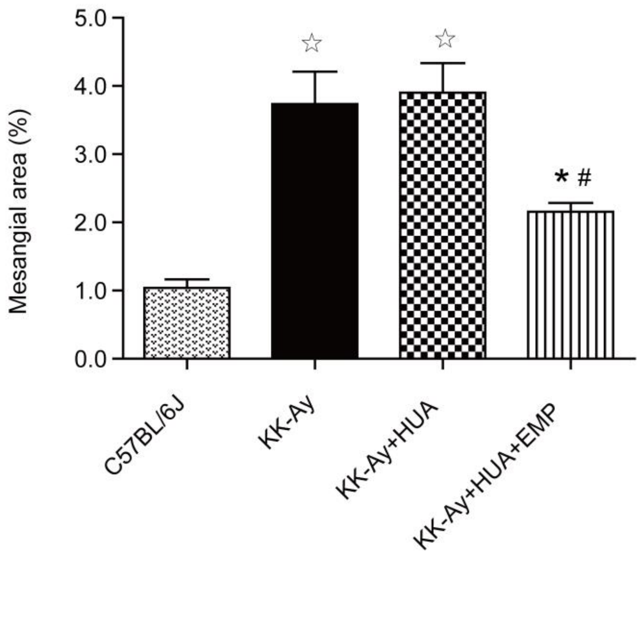

86

Supplemental figure 4

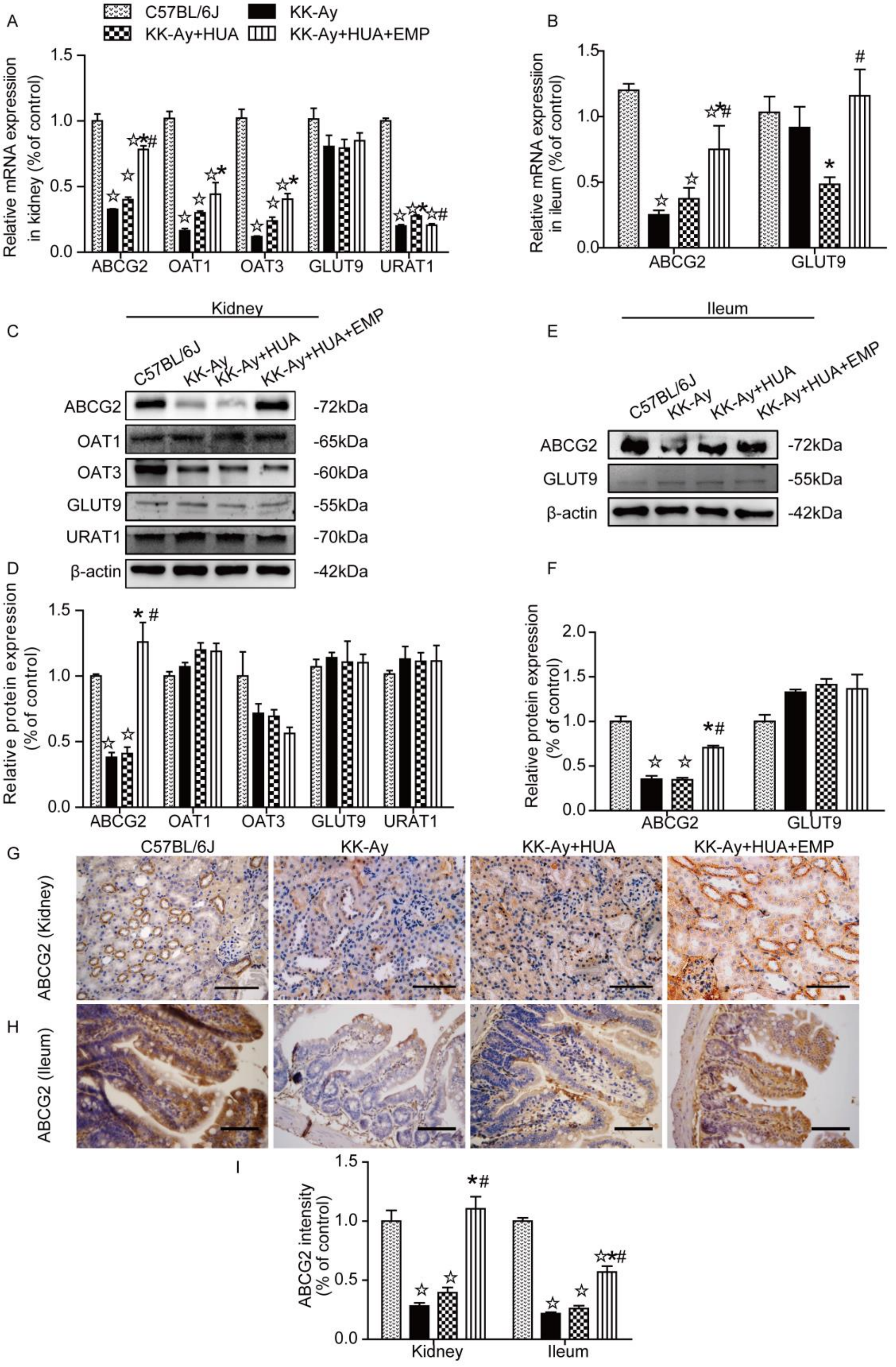

Supplemental figure 5

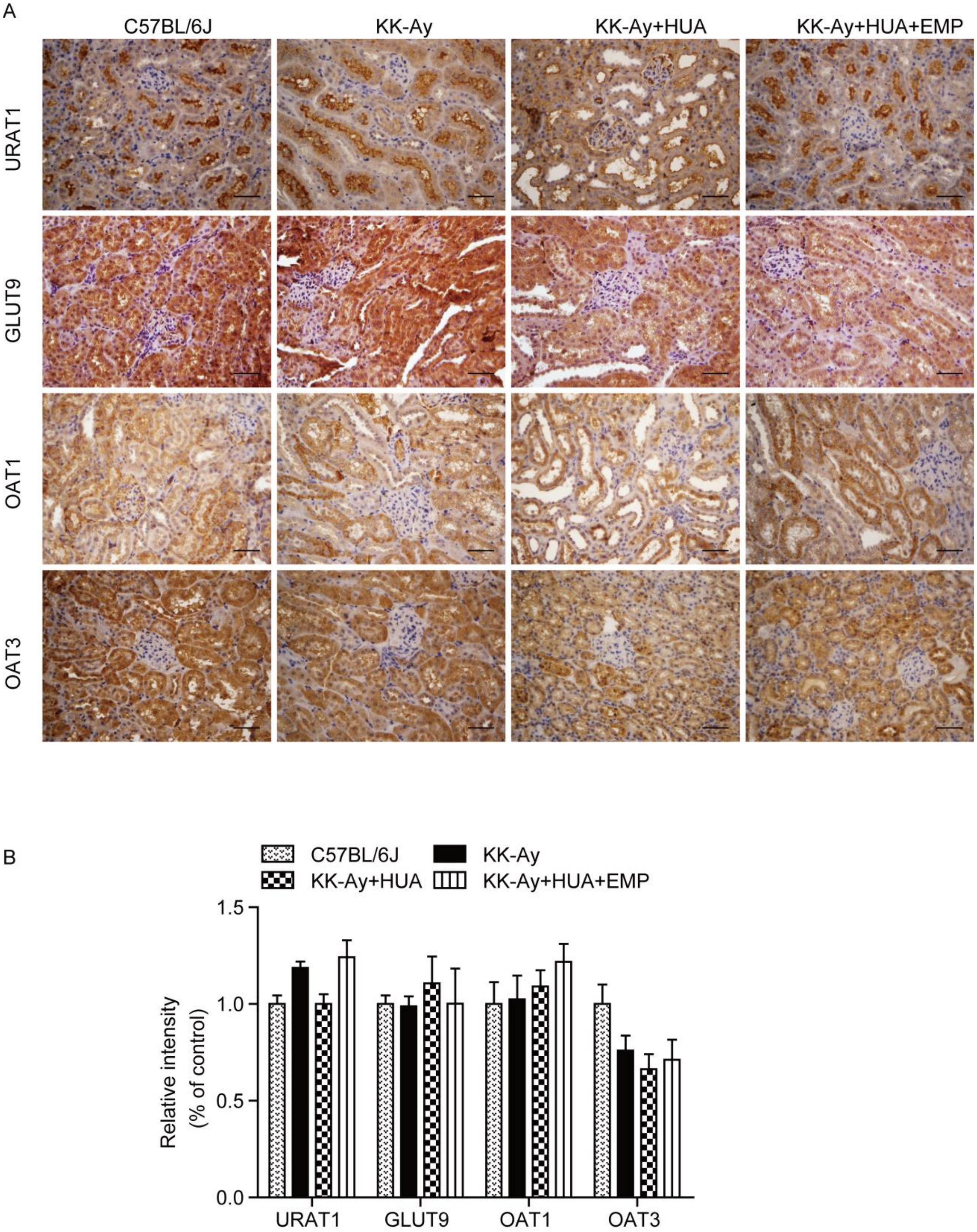

Supplemental figure 6

A

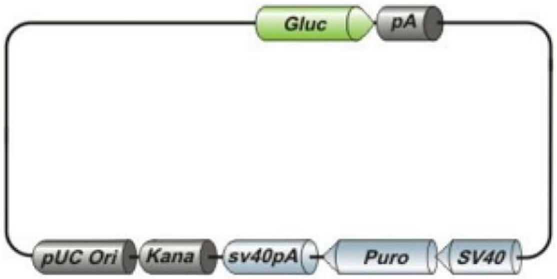

ABCG2NC

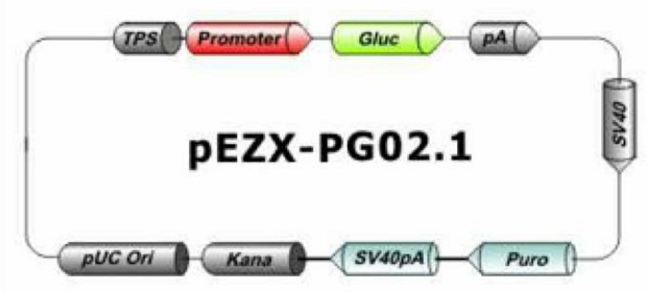

ABCG2

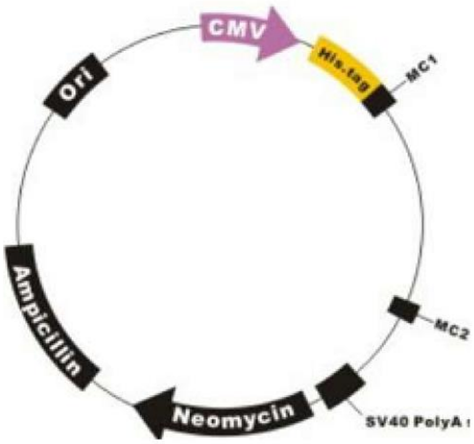

CREBNC

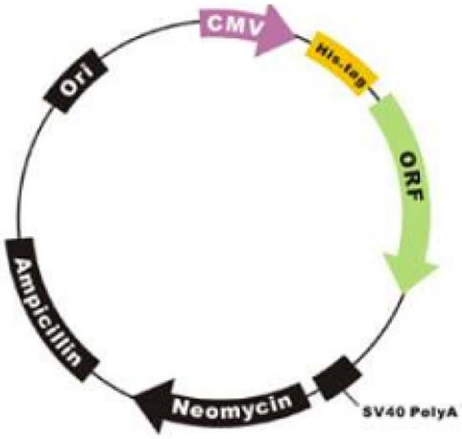

CREB
